# Serum biomarkers reflecting progression of knee osteoarthritis: a stage-based evaluation of TGF-β3, ADAMTS-4, ADAMTS-5, and MMP-13

**DOI:** 10.1186/s12891-026-09613-3

**Published:** 2026-02-14

**Authors:** Omer Esmez, Zubeyde Ercan, Secil Yılmaz, Meryem Sedef Doğru

**Affiliations:** 1 Department of Orthopedics and Traumatology, Elazığ Fethi Sekin City Hospital, Elazığ, Türkiye; 2 Department of Physiotherapy and Rehabilitation, Faculty of Health Sciences, Fırat University, Elazığ, Türkiye; 3 Department of Physiology, Faculty of Medicine, Fırat University, Elazığ, Türkiye

**Keywords:** Knee osteoarthritis, Serum biomarkers, ADAMTS-4, ADAMTS-5, MMP-13, TGF-β3, Kellgren–Lawrence grading

## Abstract

**Background:**

Knee osteoarthritis (KOA) is a progressive degenerative joint disease characterized by extracellular matrix (ECM) degradation and dysregulated inflammatory signaling. Identifying serum biomarkers associated with radiographic severity may improve early diagnosis, staging, and monitoring of progression.

**Objective:**

To evaluate stage-dependent and non-linear changes in serum transforming growth factor-β3 (TGF-β3), ADAMTS-4, ADAMTS-5, and matrix metalloproteinase-13 (MMP-13) across Kellgren–Lawrence (KL) grades of KOA.

**Methods:**

In this cross-sectional study, 200 participants were classified into five groups according to KL grade (KL0–KL4; *n* = 40 per group). Individuals with systemic inflammatory, metabolic, autoimmune, renal, cardiovascular, or bone metabolism–related disorders, and those using medications affecting cartilage or bone metabolism, were excluded. After overnight fasting, serum TGF-β3, ADAMTS-4, ADAMTS-5, and MMP-13 levels were measured by ELISA. Between-group comparisons were performed using one-way ANOVA or Kruskal–Wallis tests with appropriate post-hoc analyses, and data are presented as mean ± standard deviation, unless otherwise stated.

**Results:**

ADAMTS-4 differed significantly across KL grades (overall *p* < 0.00001), showing a non-linear pattern with higher levels in KL1–KL3. ADAMTS-5 demonstrated a pronounced stage-dependent pattern (overall *p* < 0.00002), peaking in KL1 and declining toward advanced stages. Serum MMP-13 levels did not differ significantly across KL grades (*p* > 0.05). TGF-β3 levels differed significantly across KL grades (overall *p* = 0.0015), with lower levels in KL1–KL2 compared with KL0 and intermediate levels in KL3–KL4.

**Conclusions:**

ADAMTS-4 and ADAMTS-5 exhibit biphasic, stage-dependent changes during KOA progression, consistent with early enzymatic activation followed by late-stage reduction. Early declines in TGF-β3 may reflect impaired cartilage homeostasis. The observed non-linear, stage-dependent serum patterns—particularly for ADAMTS-4, ADAMTS-5, and TGF-β3—highlight dynamic extracellular matrix remodeling during KOA progression and support the potential value of serum biomarkers as adjuncts to radiographic staging, especially in early disease.

## Introduction

Knee osteoarthritis (KOA) is the most prevalent degenerative joint disease worldwide and a leading cause of chronic pain, functional limitation, and reduced quality of life in aging populations [1, 2]. Rather than being confined solely to articular cartilage, KOA is now recognized as a disease of the whole joint, involving subchondral bone, synovium, ligaments, menisci, and periarticular musculature [3, 4].

Radiographically, KOA is characterized by joint space narrowing, osteophyte formation, subchondral sclerosis, and cystic changes; however, these features typically reflect relatively advanced disease stages [5]. Consequently, there is growing interest in identifying biological markers capable of detecting early pathological changes and monitoring disease progression before irreversible structural damage becomes evident.

A hallmark of KOA is the imbalance between anabolic and catabolic pathways within the extracellular matrix (ECM) of hyaline cartilage. The ECM is primarily composed of type II collagen fibers, which provide tensile strength, and aggrecan aggregates, which confer compressive resilience. Disruption of this matrix is initiated and accelerated by proteolytic enzymes, particularly matrix metalloproteinases (MMPs) and aggrecanases [6]. Among these, MMP-13 is recognized as the principal collagenase responsible for type II collagen degradation, while ADAMTS-4 and ADAMTS-5 are the predominant aggrecanases mediating aggrecan cleavage [7, 8]. Excessive activation of these enzymes has been consistently linked to cartilage erosion, ECM fragmentation, and subsequent subchondral bone remodeling—central events in KOA pathogenesis [6–8].

Transforming growth factor-β3 (TGF-β3), a multifunctional member of the TGF-β superfamily, plays a crucial role in maintaining cartilage homeostasis by regulating chondrocyte proliferation, differentiation, matrix synthesis, and survival. Under physiological conditions, TGF-β3 supports anabolic activity and preserves cartilage integrity; however, dysregulation of TGF-β signaling has been implicated in aberrant tissue remodeling, chondrocyte senescence, and progression toward degenerative phenotypes [9, 10]. Importantly, accumulating evidence suggests that TGF-β signaling may exert stage-dependent effects during osteoarthritis progression.

Despite extensive investigation into individual molecular mediators, there remains a lack of integrated, stage-specific serum biomarker profiles that reliably correspond to radiographic severity in KOA. Current diagnostic approaches largely rely on plain radiographs and clinical symptoms, which may inadequately reflect ongoing biochemical changes within the joint [11]. Evaluating serum biomarkers across the well-established Kellgren–Lawrence (KL) grading system may enhance diagnostic precision, identify patients at risk for rapid progression, and facilitate earlier therapeutic intervention [12].

The present study addresses this translational gap by focusing not on single biomarkers in isolation, but on the simultaneous assessment of key anabolic and catabolic mediators across the full radiographic spectrum (KL0–KL4). By evaluating ADAMTS-4 and ADAMTS-5 together, we aimed to capture potentially divergent temporal roles in aggrecanolysis, while simultaneously assessing a collagenolytic enzyme (MMP-13) and an anabolic regulatory factor (TGF-β3). We hypothesized that these biomarkers would demonstrate distinct, non-linear, stage-dependent serum patterns rather than uniform changes with increasing disease severity.

Although these biomarkers have been individually examined in KOA, to our knowledge, no previous study has comprehensively evaluated serum TGF-β3, ADAMTS-4, ADAMTS-5, and MMP-13 together across all KL grades within a balanced cohort using a unified analytical approach. Assessing these markers concurrently may provide a more holistic view of ECM remodeling dynamics and improve molecular stratification of KOA, particularly in early disease stages where radiographic findings may underestimate biochemical activity.

Therefore, the present study aimed to investigate the association between radiographic severity of knee osteoarthritis and serum levels of TGF-β3, ADAMTS-4, ADAMTS-5, and MMP-13 across KL grades 0–4, and to determine their potential utility in staging and monitoring KOA progression.

Unlike previous studies evaluating individual biomarkers in isolation, the present study simultaneously assessed anabolic (TGF-β3) and catabolic (ADAMTS-4, ADAMTS-5, MMP-13) serum biomarkers across all Kellgren–Lawrence grades within a balanced cohort. This integrative, stage-based approach allowed identification of distinct non-linear and biomarker-specific patterns rather than assuming uniform progression with radiographic severity.

## Materials and methods

### Study design and setting

This cross-sectional observational study was conducted at the Orthopedics and Traumatology outpatient clinic of Elazığ Fethi Sekin City Hospital. Participants were consecutively recruited between September 2025 and December 2025, following approval from the institutional ethics committee. Participants presenting with knee pain were clinically evaluated and screened for knee osteoarthritis according to standardized clinical and radiographic criteria.

### Radiographic assessment

Standard weight-bearing anteroposterior and lateral knee radiographs were obtained for all participants. Radiographic severity of knee osteoarthritis was assessed using the Kellgren–Lawrence (KL) classification system, which is based on four key features: joint space narrowing, osteophyte formation, subchondral sclerosis, and subchondral cyst formation [12].

KL grades were defined as follows:

- Grade 0: No radiographic features of osteoarthritis
- Grade 1: Possible osteophyte formation with doubtful joint space narrowing
- Grade 2: Definite osteophyte formation with possible joint space narrowing
- Grade 3: Moderate joint space narrowing, multiple osteophytes, and subchondral sclerosis
- Grade 4: Severe joint space narrowing, large osteophytes, marked sclerosis, and bony deformity

A total of 200 participants were enrolled and equally allocated into five groups according to KL grade (KL0–KL4; *n* = 40 per group). Radiographic evaluations were performed independently by two experienced orthopedic surgeons who were blinded to the clinical and biochemical data. Any discrepancies in grading were resolved by consensus. Interobserver agreement for KL grading was assessed using Cohen’s kappa coefficient and demonstrated excellent reliability (κ = 0.86).

### Inclusion and exclusion criteria

Participants aged 40 years and older with clinical symptoms suggestive of knee osteoarthritis and available weight-bearing knee radiographs suitable for Kellgren–Lawrence grading were eligible for inclusion. All participants provided written informed consent.

To minimize potential confounding effects on circulating biomarker levels, individuals with systemic conditions known to substantially influence inflammation, bone, or cartilage metabolism were excluded. These included uncontrolled diabetes mellitus, uncontrolled hypertension, clinically significant cardiovascular disease requiring active treatment, chronic kidney disease, autoimmune or inflammatory rheumatologic disorders, active infection or systemic inflammatory conditions, history of major lower limb trauma or prior knee surgery, and current or recent use of medications affecting bone or cartilage metabolism (e.g., systemic corticosteroids, hormone replacement therapy, thyroid hormone therapy, bisphosphonates, or disease-modifying antirheumatic drugs).

### Demographic and clinical data collection

Demographic and clinical data, including age, sex, height, weight, body mass index (BMI), symptom duration, and physical examination findings, were obtained from hospital electronic medical records and structured patient interviews.

### Blood sampling and laboratory procedures

All participants fasted for at least 8 h, and venous blood samples were collected between 08:00 and 10:00 a.m. Approximately 5 mL of venous blood was drawn. Serum was obtained by allowing blood to clot for 2 h at room temperature, followed by centrifugation at 2862 × g for 5 min.

All samples were visually inspected for hemolysis, and any samples displaying visible discoloration were excluded. Samples were aliquoted and stored at − 80 °C to prevent degradation from repeated freeze–thaw cycles. According to the manufacturer’s validation data, all analytes were stable for up to three freeze–thaw cycles; therefore, all samples were thawed only once before analysis.

#### Biomarker measurement

Serum TGF-β3, ADAMTS-4, ADAMTS-5, and MMP-13 levels were quantified using commercial human ELISA kits (Feiyue Biotechnology Co., China; catalog numbers: TGF-β3, FY-EH5632; ADAMTS-4, FY-RH1574; ADAMTS-5, FY-EH4267; MMP-13, FY-EH4375) according to the manufacturer’s instructions. The assays employed a quantitative sandwich enzyme immunoassay technique. The reported detection ranges were 15.63–1000 pg/mL for TGF-β3 and MMP-13, 62.5–4000 pg/mL for ADAMTS-4, and 0.79–50 ng/mL for ADAMTS-5. All measurements were performed in duplicate, and mean values were used for analysis. The intra-assay coefficients of variation were ≤ 10% for all kits, and no samples were below the lower limit of detection (LLOD). According to the manufacturer, the assays for ADAMTS-4, ADAMTS-5, and MMP-13 quantify total protein concentrations, including both latent (pro-) and active forms; therefore, the results reflect circulating protein burden rather than enzymatic activity. Manufacturer-provided validation indicated acceptable analytical performance for serum samples, including dilution linearity and spike–recovery within the recommended ranges.

### Statistical analysis

Statistical analyses were performed using SPSS version 22.0 (IBM Corp., Armonk, NY, USA). Data normality was assessed using the Kolmogorov–Smirnov test. Normally distributed variables were analyzed using one-way analysis of variance (ANOVA) with Tukey post-hoc tests, whereas non-normally distributed variables were compared using the Kruskal–Wallis test with Dunn post-hoc correction. Continuous variables were expressed as mean ± standard deviation (SD), and a p value < 0.05 was considered statistically significant.

To account for between-group differences in age, sex, and BMI, additional covariate-adjusted analyses were performed using a general linear model (GLM/ANCOVA), with KL grade as the fixed factor and age, sex, and BMI as covariates. Model assumptions were evaluated using residual diagnostics, and log transformation was applied when necessary. Adjusted pairwise comparisons were conducted with appropriate multiplicity correction.

A post-hoc power analysis was performed based on the primary outcome variables. With a total sample size of 200 participants and five groups (*n* = 40 each), the study achieved > 90% power to detect medium effect sizes (f = 0.25) at an alpha level of 0.05.

## Results

### Study population and KL-grade distribution

A total of 200 participants were included in the final analysis. According to radiographic severity, patients were classified into five Kellgren–Lawrence (KL) groups: KL 0 (*n* = 40), KL 1 (*n* = 40), KL 2 (*n* = 40), KL 3 (*n* = 40), and KL 4 (*n* = 40).

Baseline demographic and anthropometric characteristics stratified by KL grade are presented in Table 1. As expected, age and BMI increased progressively with higher KL grades (overall *p* < 0.001 for both). Sex distribution did not differ significantly across KL grades (*χ²*, *p* > 0.05).

**Table 1:** Demographic and clinical characteristics of the study population according to Kellgren–Lawrence (KL) grade. (Values are mean ± SD or n/%; BMI calculated as kg/m². Group comparisons by one-way ANOVA or Kruskal–Wallis test; sex by χ² test.)

Variable	KL 0 (*n* = 40)	KL 1 (*n* = 40)	KL 2 (*n* = 40)	KL 3 (*n* = 40)	KL 4 (*n* = 40)	*p* value
Age (years)	44.8 ± 4.8	52.3 ± 9.6	51.8 ± 8.9	66.4 ± 8.1	69.6 ± 6.1	< 0.001
Sex (F/M) (%)	16/24 (40/60)	28/12 (70/30)	26/14 (65/35)	28/12 (70/30)	26/14 (65/35)	0.18
BMI (kg/m²)	24.1 ± 2.9	28.9 ± 3.4	29.6 ± 4.1	31.8 ± 3.7	33.4 ± 4.0	< 0.001

Values are presented as mean ± standard deviation or number (%)

Continuous variables were compared using one-way ANOVA (or Kruskal–Wallis test where appropriate). Sex distribution was compared using the χ² test

*BMI* Body mass index, *KL* Kellgren–Lawrence

### Stage-dependent changes in serum biomarkers across KL grades

**Fig. 1:**
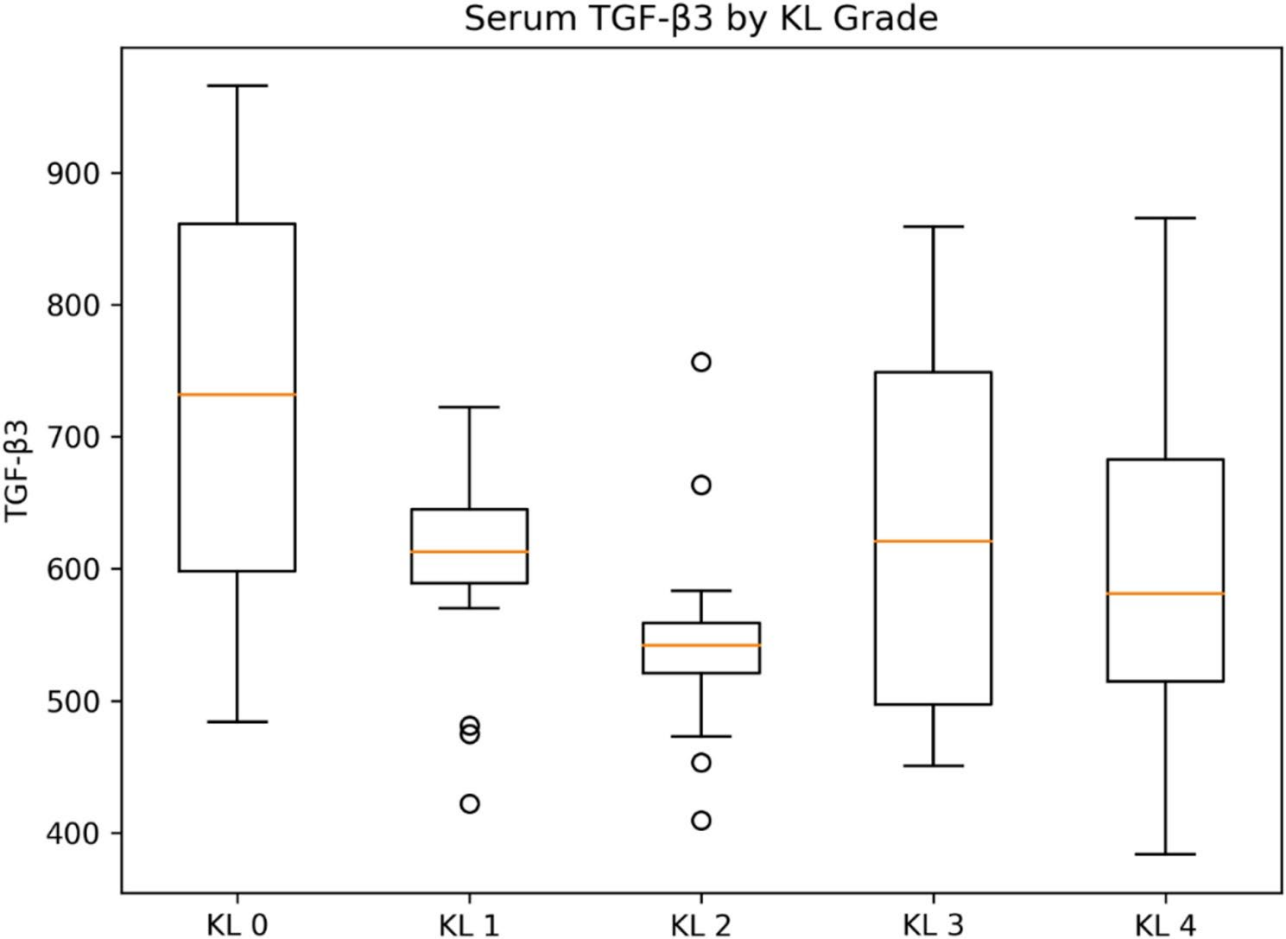
Serum TGF-β3 concentrations according to Kellgren–Lawrence (KL) grades. Box plots illustrate serum TGF-β3 (pg/mL) concentrations across Kellgren–Lawrence (KL) grades (KL0–KL4). The central line represents the median, the box indicates the Interquartile range (IQR), and whiskers extend to the most extreme values within 1.5×IQR; values beyond the whiskers are plotted as outliers. Overall group differences were significant (*p* = 0.0015). Post-hoc analysis showed significantly higher TGF-β3 levels in KL0 compared with KL1 and KL2 (*p* < 0.05)

**Fig. 2:**
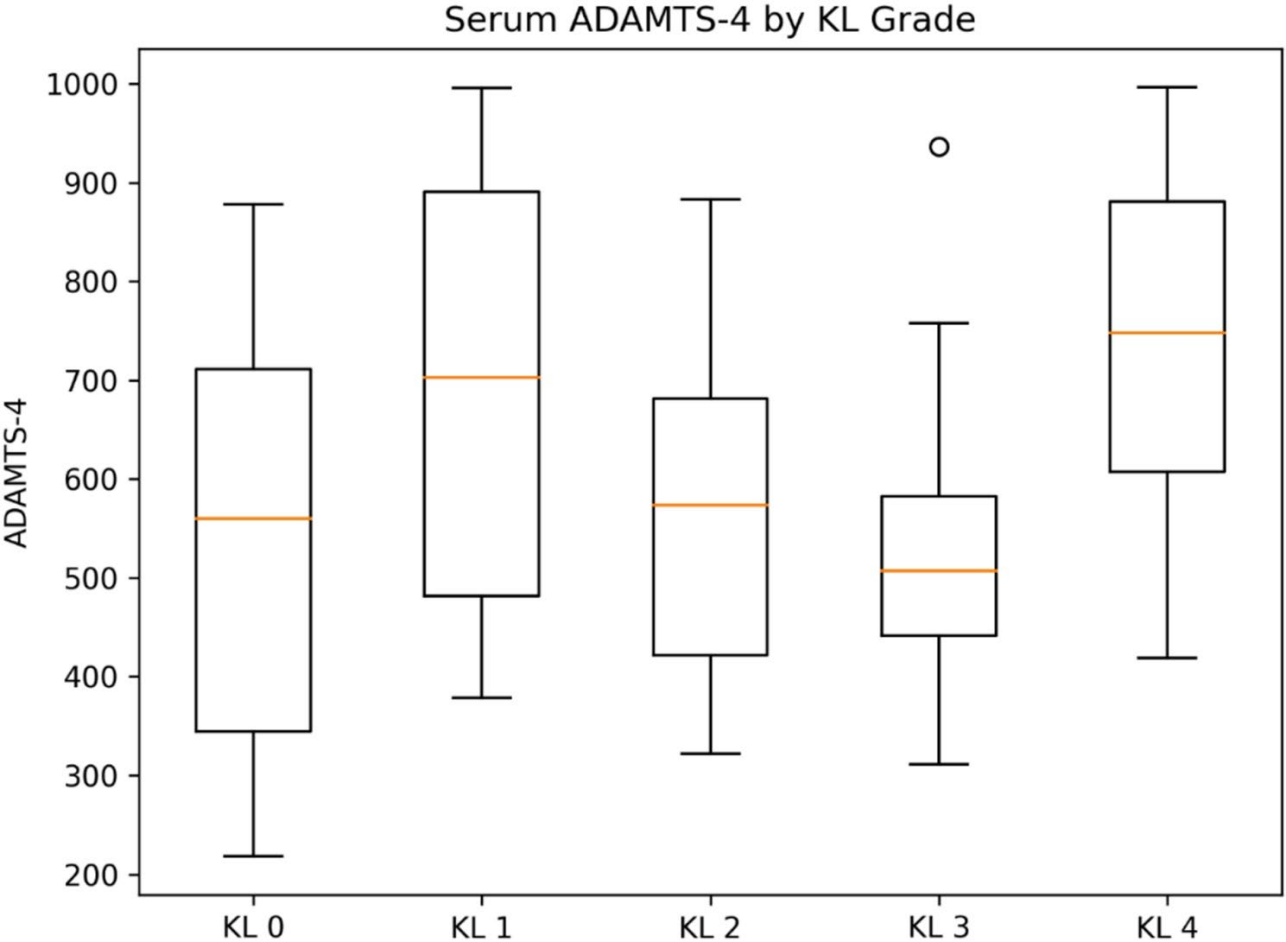
Serum ADAMTS-4 concentrations according to Kellgren–Lawrence (KL) grades. Box plots show serum ADAMTS-4 (pg/mL) concentrations stratified by KL grade. The central line represents the median, the box the IQR, whiskers extend to 1.5×IQR, and outliers are shown as individual points. Serum ADAMTS-4 levels differed significantly across KL grades (overall *p* < 0.00001), showing a stage-dependent pattern characterized by higher levels in KL1–KL3 and a decline in KL4. Post-hoc comparisons indicated significant differences between KL0 and KL1–KL3, as well as between KL3 and KL4 (*p* < 0.05)

**Fig. 3:**
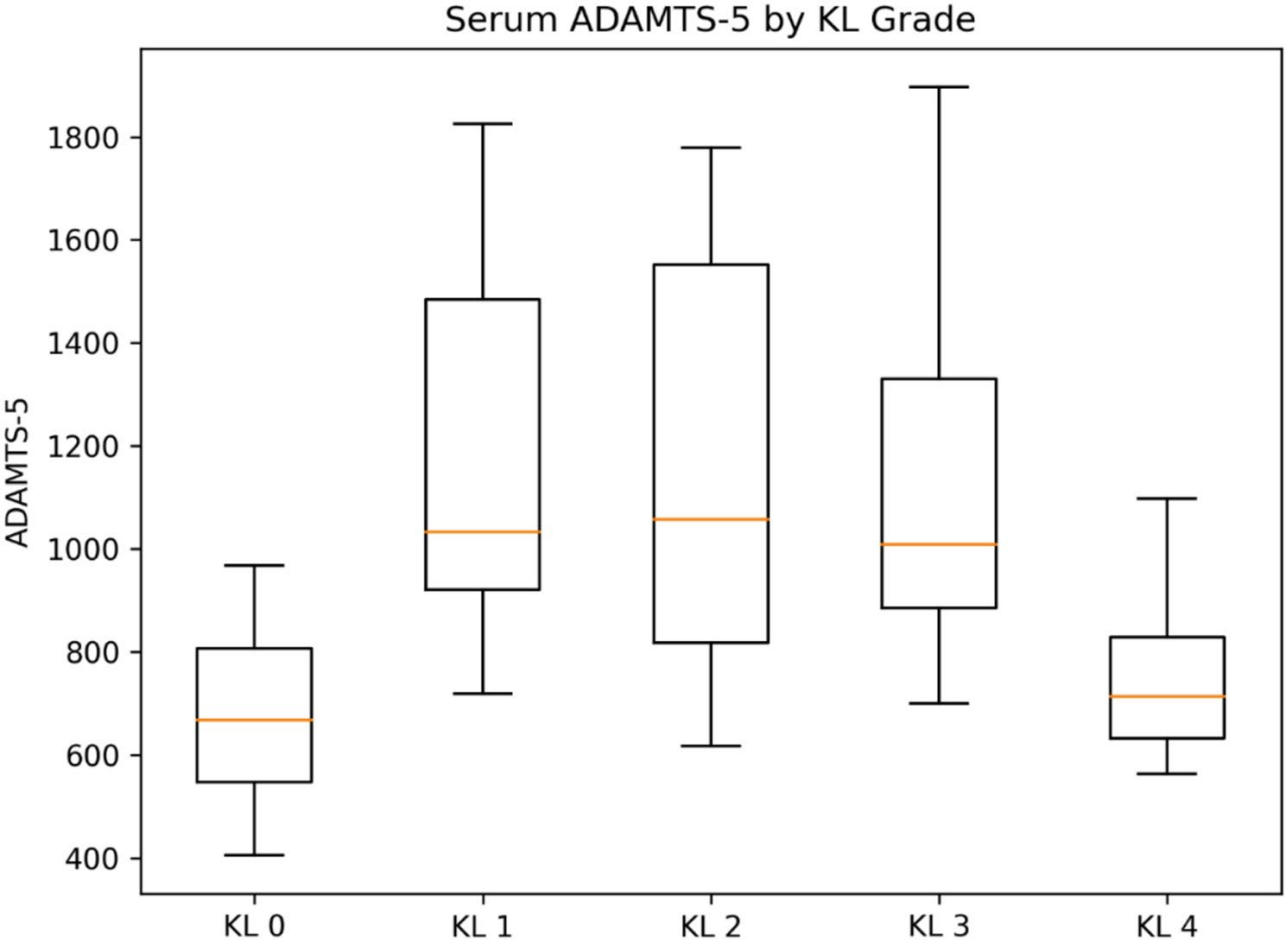
Serum ADAMTS-5 concentrations according to Kellgren–Lawrence (KL) grades. Box plots depict serum ADAMTS-5 (ng/mL) concentrations across KL grades. The median, IQR, and whiskers extending to 1.5×IQR are shown; values outside this range are plotted as outliers. A pronounced stage-dependent pattern was observed (overall *p* < 0.00002), with peak ADAMTS-5 levels in KL1 followed by a progressive decrease toward advanced radiographic stages. Post-hoc comparisons demonstrated significant differences between KL1 and KL3–KL4, and between KL2 and KL4 (*p* < 0.05)

**Fig. 4:**
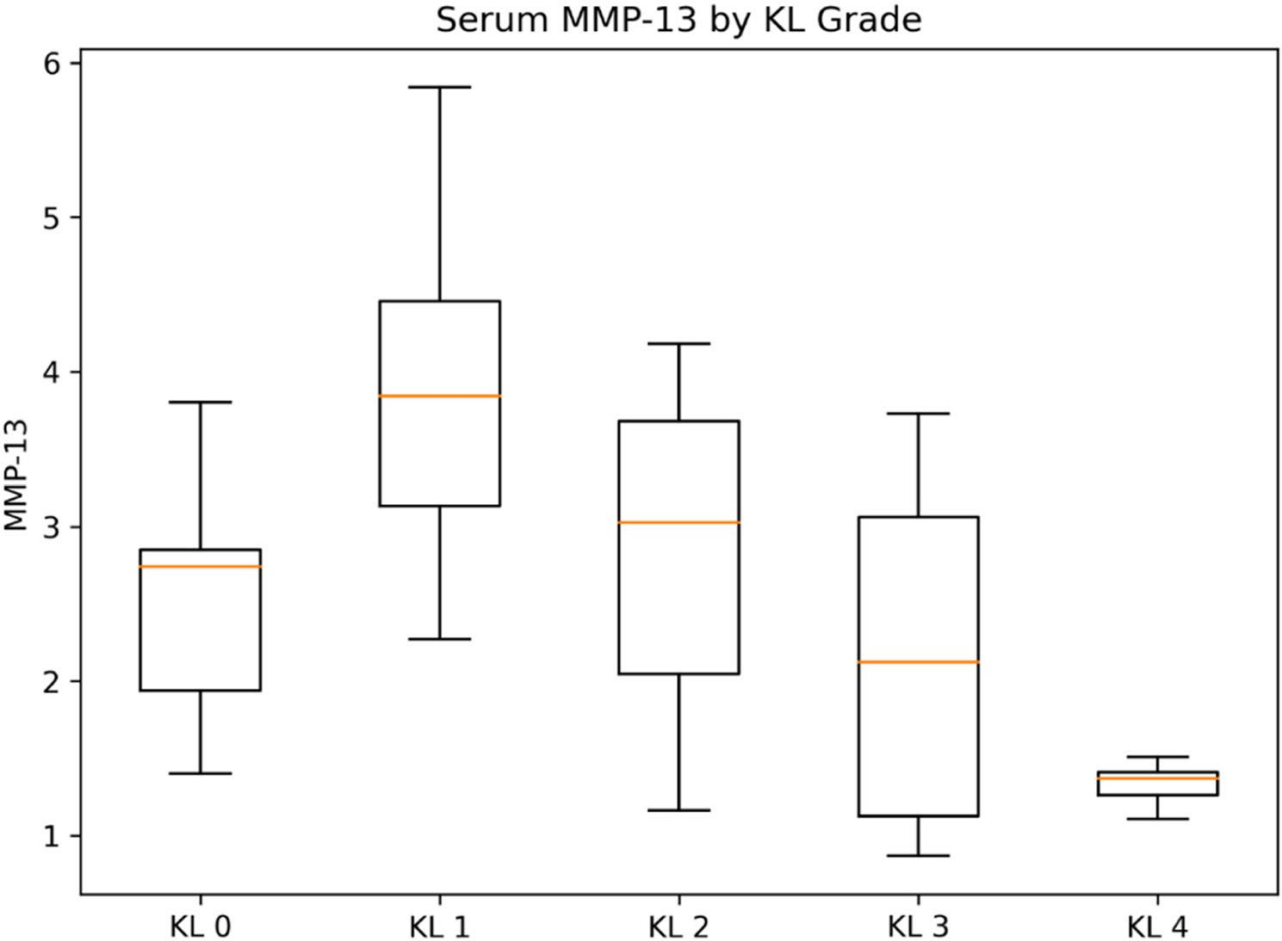
Serum MMP-13 concentrations according to Kellgren–Lawrence (KL) grades. Box plots present serum MMP-13 (pg/mL) concentrations according to KL grade. The central line represents the median, the box the IQR, whiskers extend to 1.5×IQR, and outliers are shown as individual points. Serum MMP-13 levels did not differ significantly across KL grades (overall *p* > 0.05), indicating that circulating MMP-13 may be less sensitive to radiographic stage in this cross-sectional cohort

Serum concentrations of TGF-β3, ADAMTS-4, and ADAMTS-5 differed significantly across KL grades, whereas MMP-13 did not show a statistically significant difference between groups (overall tests below). Group-wise distributions are shown as box plots (Figs. 1, 2, 3 and 4).

Normality assessment using the Kolmogorov–Smirnov test demonstrated that serum TGF-β3 and ADAMTS-5 levels were normally distributed across KL grades, whereas ADAMTS-4 and MMP-13 showed non-normal distributions. Accordingly, one-way ANOVA with Tukey post-hoc test was used for TGF-β3 and ADAMTS-5, while the Kruskal–Wallis test followed by Dunn’s post-hoc correction was applied for ADAMTS-4 and MMP-13. Tables present data as mean ± SD, whereas box plots display medians and interquartile ranges.

#### TGF-β3

Mean serum TGF-β3 levels differed across KL grades (overall *p* = 0.0015). TGF-β3 was highest in KL0 and lower in KL1–KL2, with intermediate levels in KL3–KL4. Pairwise comparisons were performed using Tukey’s post-hoc test.

#### ADAMTS-4

Serum ADAMTS-4 levels differed significantly across KL grades (overall *p* < 0.00001), demonstrating a non-linear, stage-dependent pattern. Levels increased markedly from KL0 to KL1–KL3 and declined in KL4. The relatively low variability observed in the KL3 group likely reflects a more homogeneous biochemical profile at this intermediate–advanced stage within the balanced cohort, rather than a methodological or analytical artifact.

#### ADAMTS-5

Serum ADAMTS-5 levels showed a pronounced stage-dependent pattern (overall *p* < 0.00002), peaking in KL1 and progressively decreasing toward advanced stages.

#### MMP-13

Serum MMP-13 levels did not differ significantly across KL grades (overall *p* > 0.05).

### Summary of biomarker concentrations by KL grade

Table 2 summarizes serum biomarker concentrations by KL grade (mean ± SD). The table demonstrates distinct stage-dependent patterns for TGF-β3, ADAMTS-4, and ADAMTS-5, whereas MMP-13 does not show a statistically significant stage-dependent difference.

**Table 2:** Serum biomarker concentrations according to Kellgren–Lawrence (KL) grade

Biomarker	KL 0	KL 1	KL 2	KL 3	KL 4	Overall *p*
TGF-β3 (pg/mL)	729.1 ± 153.9	604.8 ± 86.3	546.5 ± 80.0	623.8 ± 137.5	590.1 ± 128.8	0.0015
ADAMTS-4 (pg/mL)	672.3 ± 185.3	1192.0 ± 373.1	1161.1 ± 405.8	1106.2 ± 43.9	746.5 ± 148.1	< 0.00001
ADAMTS-5 (ng/mL)	2.56 ± 0.81	3.88 ± 1.03	2.82 ± 1.08	2.19 ± 1.05	1.33 ± 0.14	< 0.00002
MMP-13 (pg/mL)	540.8 ± 228.2	692.4 ± 214.4	561.1 ± 160.2	537.5 ± 154.7	721.6 ± 186.5	>0.05

Values are presented as mean ± standard deviation. Overall p values were obtained using one-way ANOVA (TGF-β3, ADAMTS-5) or Kruskal–Wallis test (ADAMTS-4, MMP-13), according to distribution

### Sex-based subgroup analysis

To address potential sex-related differences, sex-stratified analyses were performed for all four biomarkers. No significant differences were observed between male and female patients for TGF-β3, ADAMTS-4, ADAMTS-5, or MMP-13 across KL stages (all *p* > 0.05).

Furthermore, no significant sex × KL interaction was detected for any biomarker, indicating that the observed stage-dependent changes were independent of sex.

The absence of significant sex-related differences in serum biomarker levels should be interpreted with caution. While sex hormones and biomechanical factors may influence local joint pathology, systemic serum concentrations reflect an integrated biological pool in which localized intra-articular differences may be attenuated. Moreover, sex-related effects in osteoarthritis may preferentially manifest at the tissue or synovial fluid level rather than in circulating biomarkers, particularly in cross-sectional analyses.

### Multivariate analysis

In multivariate models adjusted for age, BMI, and sex, KL grade remained an independent determinant of serum biomarker levels. The association between KL grade and ADAMTS-5 remained significant after adjustment, whereas TGF-β3 demonstrated an independent association with lower KL stages. No independent association was observed between KL grade and MMP-13 after adjustment (*p* > 0.05).

### Key findings

Overall, this study demonstrates that:

1. Serum levels of TGF-β3, ADAMTS-4, and ADAMTS-5 vary significantly across radiographic stages of knee osteoarthritis, whereas MMP-13 does not show a statistically significant stage-dependent difference.
2. ADAMTS-5 exhibits the most pronounced stage-dependent increase, peaking in mild-to-moderate disease.
3. Sex does not modify the relationship between KL grade and serum biomarker levels.

## Discussion

The novelty of this study lies not in the identification of new biomarkers, but in the integrated, stage-specific interpretation of anabolic–catabolic imbalance across the full radiographic spectrum of knee osteoarthritis using a uniform analytical framework. By simultaneously evaluating key anabolic and catabolic serum biomarkers—TGF-β3, ADAMTS-4, ADAMTS-5, and MMP-13—within a balanced cohort spanning all Kellgren–Lawrence (KL) grades, we provide a comprehensive view of extracellular matrix (ECM) remodeling dynamics during KOA progression.

Osteoarthritis is increasingly recognized as a whole-joint disease involving coordinated pathological processes in articular cartilage, subchondral bone, synovium, and periarticular tissues rather than isolated cartilage degeneration [1, 2, 4]. In this context, identifying circulating biomarkers that reflect disease stage and underlying molecular activity remains a major research priority, particularly for early disease where radiographic changes may be subtle or absent [5, 11].

### Aggrecanases and stage-dependent ECM remodeling

A principal finding of the present study is the non-linear, stage-dependent behavior of serum aggrecanases, particularly ADAMTS-4 and ADAMTS-5. Aggrecan degradation represents an early and critical molecular event in KOA pathogenesis, preceding irreversible disruption of the collagen network [6, 8]. Consistent with this concept, serum ADAMTS-5 demonstrated a pronounced increase from KL0 to KL1–KL3, followed by a decline in KL4, indicating peak aggrecanase activity during mild-to-moderate radiographic disease. This pattern suggests that ADAMTS-5 may play a dominant role in early aggrecanolysis when cartilage metabolism remains active and chondrocyte-driven matrix turnover predominates.

In contrast, ADAMTS-4 exhibited a distinct non-linear profile, with higher levels observed in KL1–KL3 followed by a decline in KL4. Rather than reflecting a simple monotonic relationship with radiographic severity, this pattern implies stage-specific regulation by different biological mechanisms. Elevated ADAMTS-4 levels in earlier-to-intermediate stages may correspond to active aggrecan degradation, whereas the decline in advanced KOA could reflect reduced viable cartilage, altered enzyme regulation, or diminished systemic spillover in end-stage disease. Prior experimental and clinical studies have proposed temporally and functionally distinct roles for ADAMTS-4 and ADAMTS-5, supporting the view that these enzymes are not interchangeable and contribute differently across disease stages [6, 7, 13]. Our findings reinforce this distinction and underscore the value of concurrent assessment of multiple aggrecanases rather than reliance on a single catabolic marker.

### Collagen degradation and MMP-13

Serum MMP-13 levels did not differ significantly across KL grades (overall *p* > 0.05). Although MMP-13 is widely recognized as a principal collagenase responsible for type II collagen degradation in osteoarthritic cartilage [4, 8], circulating MMP-13 may be less sensitive to radiographic staging than aggrecanases in a cross-sectional setting. Therefore, serum MMP-13 should be considered a complementary marker rather than a standalone indicator of disease severity.

The lack of a significant stage-dependent difference in circulating MMP-13 does not preclude its established role in cartilage collagen degradation. MMP-13 activity is predominantly localized within the joint microenvironment, and serum measurements—particularly when quantifying total protein rather than enzymatic activity—may underestimate localized collagenolytic processes. Moreover, collagen network disruption typically represents a later and more spatially confined event in osteoarthritis progression, which may not be adequately captured by systemic cross-sectional assessment.

### Anabolic regulation and TGF-β3

Another important observation is the stage-dependent alteration in serum TGF-β3, a key anabolic regulator of cartilage homeostasis. TGF-β signaling is central to chondrocyte proliferation, differentiation, and ECM synthesis under physiological conditions [9, 10]. In this study, TGF-β3 levels were significantly lower in KL1 and KL2 compared with KL0, suggesting early impairment of anabolic signaling during the initial phases of KOA. Intermediate levels in KL3–KL4 indicate complex, non-linear regulation as disease advances. Dysregulated TGF-β signaling has been implicated in chondrocyte senescence and aberrant tissue remodeling, particularly in advanced osteoarthritis [9, 10]. Thus, reduced systemic TGF-β3 in early disease may represent an early molecular signature of compromised cartilage repair capacity preceding overt structural damage detectable by conventional imaging.

### Integrated interpretation and clinical implications

Taken together, the combined assessment of anabolic (TGF-β3) and catabolic (ADAMTS-4, ADAMTS-5, MMP-13) biomarkers provides a more nuanced understanding of ECM remodeling during KOA progression than single-marker approaches. The stage-dependent and non-linear patterns identified here suggest that serum biomarkers may have greatest clinical utility in early and intermediate disease stages, when active molecular remodeling predominates. This has important implications for patient stratification and for the development of disease-modifying osteoarthritis therapies, which are most likely to be effective when initiated before irreversible structural damage occurs [7, 14].

From a clinical perspective, the pronounced stage-dependent serum patterns of ADAMTS-4, ADAMTS-5, and TGF-β3 suggest that these biomarkers may be most informative in early and intermediate KOA, when radiographic changes may underestimate ongoing molecular activity. Such biomarker profiles could support patient stratification and selection for disease-modifying interventions at a stage when cartilage remodeling may still be biologically active.

### Limitations

Several limitations warrant consideration. The cross-sectional design precludes causal inference and limits evaluation of longitudinal biomarker dynamics. Serum biomarker levels may not fully reflect local intra-articular processes within cartilage and synovial tissue, and be entirely excluded despite strict exclusion criteria. Future longitudinal studies integrating serum, synovial fluid, and imaging biomarkers are needed to validate the prognostic and therapeutic utility of these markers.

Although synovial fluid may more directly reflect intra-articular processes, serum biomarkers were selected for their clinical feasibility, reproducibility, and potential applicability to large-scale screening and longitudinal monitoring.

## Conclusion

In conclusion, this study demonstrates that serum ADAMTS-4, ADAMTS-5, TGF-β3, and MMP-13 exhibit distinct, stage-dependent alterations across radiographic knee osteoarthritis. These findings support the concept that selected serum biomarkers may complement radiographic assessment for staging and monitoring KOA progression, particularly in early disease stages, and may inform future biomarker-guided therapeutic strategies. 

## Data Availability

The datasets generated and/or analyzed during the current study are available from the corresponding author on reasonable request.

## References

[1] Glyn-Jones S, Palmer AJR, Agricola R, et al. Osteoarthr Lancet. 2015;386(9991):376–87. 10.1016/S0140-6736(14)60802-3. 10.1016/S0140-6736(14)60802-3 25748615

[2] Hunter DJ, Bierma-Zeinstra S, Osteoarthritis. Lancet. 2019;393(10182):1745–59. 10.1016/S0140-6736(19)30417-9. 31034380 10.1016/S0140-6736(19)30417-9

[3] Li B, Yang Z, Li Y, et al. Exploration beyond osteoarthritis: the association and mechanism of its related comorbidities. Front Endocrinol (Lausanne). 2024;15:1352671. 10.3389/fendo.2024.1352671. 38779455 10.3389/fendo.2024.1352671 PMC11110169

[4] Loeser RF, Goldring SR, Scanzello CR, Goldring MB. Osteoarthritis: a disease of the joint as an organ. Arthritis Rheum. 2012;64(6):1697–707. 22392533 10.1002/art.34453 PMC3366018

[5] Katz JN, Arant KR, Loeser RF. Diagnosis and treatment of hip and knee osteoarthritis: a review. JAMA. 2020;325(6):568–78. 10.1001/jama.2020.22171. 10.1001/jama.2020.22171 PMC8225295 33560326

[6] Verma P, Dalal K. ADAMTS-4 and ADAMTS-5: key enzymes in osteoarthritis. J Cell Biochem. 2011;112(12):3507–14. 21815191 10.1002/jcb.23298

[7] Oo WM, Yu SPC, Daniel MS, Hunter DJ. Disease-modifying drugs in osteoarthritis. Expert Opin Emerg Drugs. 2018;23(4):331–47. 30415584 10.1080/14728214.2018.1547706

[8] Billinghurst RC, Dahlberg L, Ionescu M, et al. Enhanced cleavage of type II collagen by collagenases in Osteoarthritic cartilage. J Clin Invest. 1997;99(7):1534–45. 9119997 10.1172/JCI119316 PMC507973

[9] Du X, Cai L, Xie J, Zhou X. The role of TGF-β3 in cartilage development and osteoarthritis. Bone Res. 2023;11(1):2. 36588106 10.1038/s41413-022-00239-4 PMC9806111

[10] Cherifi C, Monteagudo S, Lories RJ. Promising targets for therapy of osteoarthritis: Wnt and TGF-β signalling pathways. Ther Adv Musculoskelet Dis. 2021;13:1759720X211006959. 33948125 10.1177/1759720X211006959 PMC8053758

[11] Karsdal MA, Byrjalsen I, Bay-Jensen AC, et al. Biochemical markers identify influences on bone and cartilage degradation in osteoarthritis. BMC Musculoskelet Disord. 2010;11:125. 20565725 10.1186/1471-2474-11-125 PMC2902412

[12] Kellgren JH, Lawrence JS. Radiological assessment of osteo-arthrosis. Ann Rheum Dis. 1957;16(4):494–502. 13498604 10.1136/ard.16.4.494 PMC1006995

[13] Tortorella MD, Malfait AM. Will the real aggrecanase(s) step up: evaluating the criteria that define aggrecanase activity in osteoarthritis. Curr Pharm Biotechnol. 2008;9(1):16–23. 18289053 10.2174/138920108783497622

[14] Little CB, Hunter DJ. Post-traumatic osteoarthritis: from mouse models to clinical trials. Nat Rev Rheumatol. 2013;9(8):485–97. 10.1038/nrrheum.2013.72. 23689231 10.1038/nrrheum.2013.72

